# Hemodynamics and matrix stiffness shape the pathogenicity of SPP1^+^ macrophages

**DOI:** 10.3389/fimmu.2026.1856522

**Published:** 2026-05-29

**Authors:** Hongjiu Zhang, Jiang Han, Shirong Zhu, Manhong Yi, Keyi Fan, Runze Chang, Yongbin Shi, Honglin Dong

**Affiliations:** 1 Department of Vascular Surgery, The Second Hospital of Shanxi Medical University, Taiyuan, Shanxi, China; 2 Department of Nephrology, The Second Hospital of Shanxi Medical University, Taiyuan, Shanxi, China

**Keywords:** atherosclerosis, extracellular matrix remodeling, mechanobiology, SPP1+ macrophages, YAP/TAZ

## Abstract

**Background:**

Macrophages exhibit significant plasticity and are integral to tissue homeostasis and disease progression. Recent single-cell RNA sequencing (scRNA-seq) analyses identify secreted phosphoprotein 1-positive (SPP1^+^) macrophages as a highly conserved pathogenic subpopulation. This subset predominantly localizes to the necrotic cores and calcified regions of vascular lesions, contributing to dysregulation of lipid metabolism and pathological extracellular matrix (ECM) remodeling. While traditional pathology emphasizes biochemical signaling, the local physical microenvironment, including abnormal hemodynamics and increased matrix stiffness, serves as a critical mechanical stimulus that directly mediates the transcriptional reprogramming of macrophages toward a pathogenic phenotype.

**Objective:**

This review systematically synthesizes recent advancements concerning the role of the local mechanical microenvironment in modulating the phenotypic transition of SPP1^+^ macrophages. It aims to elucidate the physical mechanisms underlying immune cell activation in vascular pathologies.

**Highlights:**

Macrophages capture external physical stimuli through a sensing network comprising integrins, Piezo-type mechanosensitive ion channel component 1 (PIEZO1), and primary cilia. Abnormal mechanical forces induce increased intracellular cytoskeletal tension, which drives the nuclear translocation of Yes-associated protein (YAP) and transcriptional coactivator with PDZ-binding motif (TAZ). The activation of these mechanosensitive transcription factors directly upregulates the expression of *SPP1*. Extracellularly secreted SPP1 subsequently mediates pathological ECM cross-linking and microcalcification, further exacerbating local matrix stiffening. Based on current literature, we propose that this cascade potentially establishes a detrimental positive mechanical feedback loop, sustaining the continuous pathogenic activation of macrophages and accelerating vascular disease progression.

**Conclusion:**

The physical microenvironment represents a critical determinant of macrophage plasticity. Developing targeted interventions directed at these mechanotransduction pathways provides a viable therapeutic strategy for the management of macrophage-mediated arterial diseases and tissue fibrosis.

## 1 Introduction

Macrophages are the core cells of the innate immune system (1). Owing to their high plasticity, they play a key role in maintaining tissue homeostasis and driving disease progression across various biological contexts (2–4). Traditionally, macrophage polarization and functional changes are thought to be driven solely by biochemical signals such as inflammatory factors or lipid mediators (5, 6). For decades, the pathological understanding of immune responses in vascular remodeling was heavily constrained by these circulating soluble cascades. However, simple biochemical cascades cannot fully explain the spatial specificity of lesions. In cardiovascular pathology, atherosclerosis usually occurs at vascular bifurcations or curvatures and rarely affects straight vascular segments (7, 8). The striking contrast between circulating systemic risk factors and localized lesion formation indicates that spatial signals synergize with classical biochemical pathways to drive macrophage activation within specific vascular segments (9).

With the wide application of scRNA-seq, the complex heterogeneity map of macrophages has been redrawn (10–12). This technological advancement has allowed researchers to move beyond simplified *in vitro* models, revealing highly specialized and dynamic cellular states *in vivo*. Among these diverse states, distinct subsets uniquely responsive to the vascular microenvironment have emerged. SPP1^+^ macrophages, in particular, have been identified as a highly conserved pathogenic subpopulation (13–15). A significant population of SPP1^+^ macrophages is localized within the necrotic core and calcified regions of the vessels. Xiong et al. revealed a preferential localization of SPP1^+^ in foamy macrophages within the hypoxic core of atherosclerotic plaques, highlighting the role of the local microenvironment (16). Furthermore, as recently comprehensively reviewed by Zhao et al., rather than merely adapting to this hypoxic niche, these macrophages actively drive structural degradation and vascular calcification (17). The discovery of this spatially restricted, highly pathogenic subset further underscores the inadequacy of relying purely on systemic biochemical signals to explain the precise focal nature of vascular lesion formation and progression.

The local physical microenvironment is a critical yet often overlooked dimension in vascular biology. In arterial lesions, this environment is defined by dynamic mechanical forces, including extracellular matrix stiffness, cyclic stretch, and shear stress. Emerging evidence from the burgeoning field of mechanoimmunology underscores that physical stimuli function as pivotal mechanical cues that modulate immune cell behavior (18, 19). Specifically, biomechanical forces such as disturbed fluid shear stress and matrix stiffness mediate the transcriptional reprogramming of macrophages (20–22). In the complex tissue microenvironment, macrophages are not passive recipients. They actively sense mechanical changes. Through various surface or intracellular mechanoreceptors, such as integrins, mechanosensitive ion channels such as PIEZO1, and primary cilia, macrophages rapidly convert external physical stimuli into intracellular biochemical cascades (23–25). These meticulously orchestrated signals eventually converge on mechanosensitive nuclear transcription factors, primarily YAP/TAZ. This dynamic mechanotransduction process significantly upregulates the expression of *SPP1* and other profibrotic or proinflammatory genes, transforming physical forces into enduring genomic responses (26).

Physical forces occupy a central role in vascular biology. Mechanotransduction pathways are extensively mapped in inherent vascular wall cells, including endothelial and smooth muscle cells (27–29). Emerging studies explicitly link localized hemodynamics and matrix stiffness to macrophage polarization (25, 30). Stiffened extracellular matrix triggers intracellular *SPP1* upregulation via mechanosensitive integrins and ion channels (24). Altered shear stress accelerates the recruitment of this pathogenic subset during vascular remodeling (31). The precise mechanism by which the mechanical microenvironment regulates this specific SPP1^+^ macrophage phenotype lacks a systematic explanation. A critical knowledge void remains in understanding this complex regulatory network.

**Figure 1:**
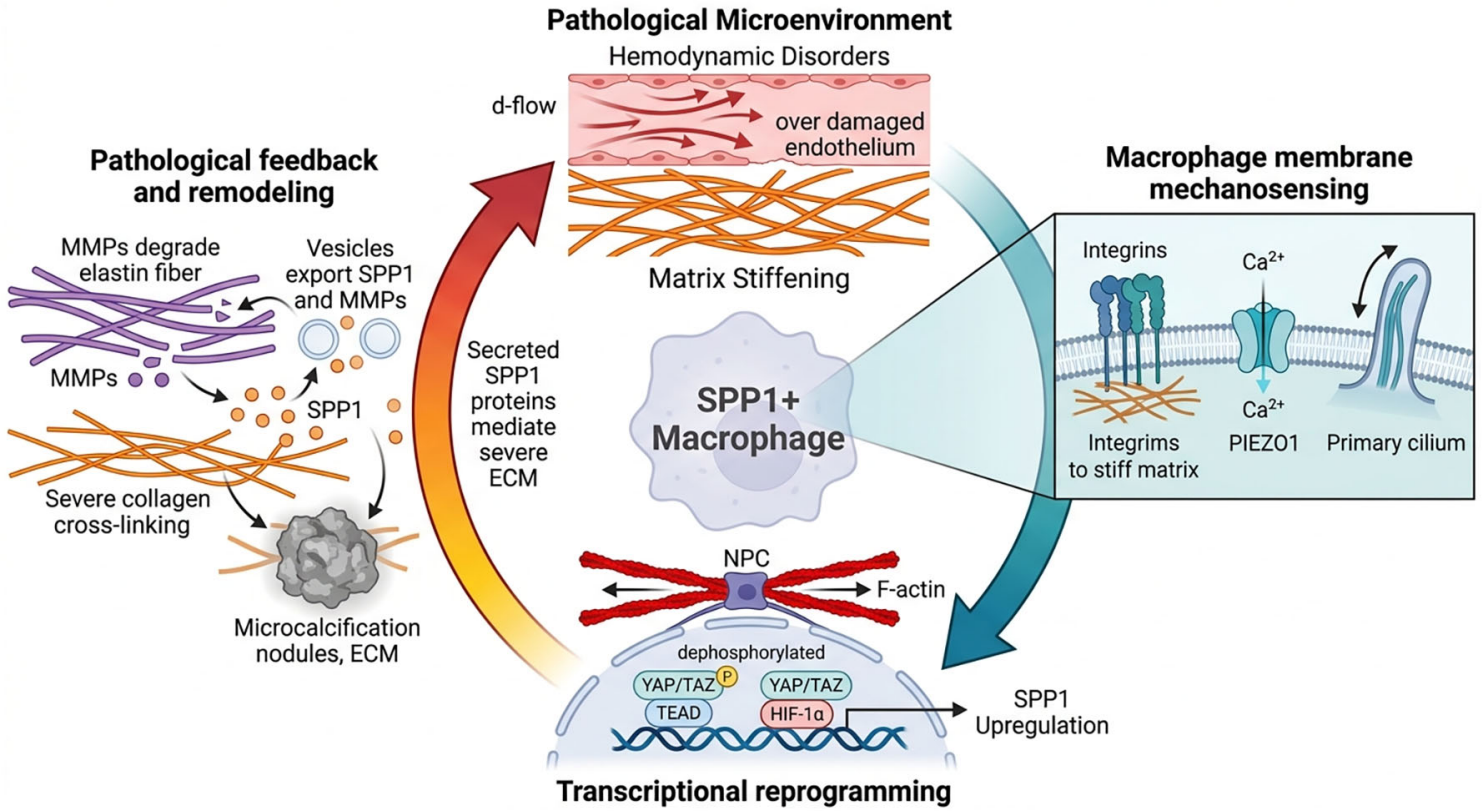
D-flow induced mechanotransduction and vascular remodeling. A pathological microenvironment induced by disturbed flow activates SPP1^+^ macrophages. Via mechanosensors like integrins and PIEZO1, these cells convert stiffness signals into YAP/TAZ/HIF-1α-driven transcriptional reprogramming, upregulating *SPP1*. This secretion activates MMPs to degrade elastin and promotes collagen cross-linking and microcalcification, establishing a pathological feedback loop that exacerbates vascular stiffening. (Created with BioGDP.com).

This article systematically reviews the latest progress in macrophage mechanobiology, focusing on the specific mechanosensors driving the SPP1^+^ phenotype transition and their dependent YAP/TAZ downstream signaling pathways. We further analyze the hypothesized pathological mechanical positive feedback loop between the microenvironment and immune cells, proposing a conceptual framework of how early extracellular matrix stiffening induced by abnormal hemodynamics sustains macrophages in a state of pathological activation. Furthermore, this review summarizes current *in vitro* biomimetic platforms and *in vivo* genetically engineered models used to validate these mechano-immune mechanisms, and explores the clinical translational prospects of targeted interventions and intelligent nanodelivery systems designed to intercept mechanotransduction pathways. By systematically depicting this complex mechanical response network, we hope to provide new mechanistic insights and therapeutic perspectives for macrophage-driven arterial diseases and tissue fibrosis. (**Figure 1**).

## 2 Mechanical stimulation of the vascular microenvironment and physical perception of macrophages

### 2.1 Hemodynamic disorders and matrix stiffening

The cardiovascular system is always in a dynamic mechanical network. Blood flow primarily transmits vertical hydrostatic pressure and parallel fluid shear stress (FSS) to the vascular wall (32). In straight vessel segments, blood flows in a regular, laminar manner. This generates unidirectional and relatively high physiological shear stress (>15 dyn/cm²). This mechanical microenvironment is fundamental for maintaining endothelial homeostasis and normal vascular wall functions (33, 34). However, in specific anatomical areas, such as arterial bifurcations, curves, or stenoses (e.g., the carotid sinus and aortic arch), local hemodynamics change significantly to form disturbed flow (d-flow). A typical feature of this flow pattern is low shear stress (usually <4 dyn/cm²), accompanied by directional oscillation and turbulence (35). This abnormal mechanical stimulus triggers proinflammatory activation and barrier damage in endothelial cells (36, 37). Beyond its profound impact on the endothelium, disturbed flow also orchestrates the behavior of macrophages colonized in the subintima, though the nature of this interaction depends heavily on the integrity of the endothelial barrier. Beneath an intact endothelial layer, luminal flow primarily influences macrophages indirectly through endothelial cell-derived paracrine signals, increased barrier permeability, and early ECM remodeling (38). However, macrophages may also sense mechanical forces directly via altered local interstitial flow or upon direct exposure to the lumen in advanced lesions with endothelial denudation (39). Through these direct and indirect mechanisms, disturbed flow acts as a key physical cue that may contribute to initiating the microenvironmental remodeling required for the transition of macrophages toward the SPP1^+^ phenotype.

In addition to dynamic fluid mechanics, the macrophage phenotype is regulated by its solid microenvironment, namely, the mechanical properties of the ECM. Physiologically, a healthy arterial wall is rich in elastin, showing good compliance and low matrix stiffness. Its intimal stiffness is usually approximately 2–5 kPa (40, 41). Under long-term risk factors such as aging, hypertension, or chronic inflammation, the vascular wall undergoes pathological remodeling. Significant changes in ECM components accompany this process (42). Abnormal matrix metalloproteinase (MMP) activity leads to extensive degradation of elastic fibers. Concurrently, smooth muscle cells and fibroblasts excessively secrete prestiffening type I and type III collagen (43, 44). Furthermore, collagen cross-linking mediated by lysyl oxidase (LOX) exacerbates matrix densification (45). These biochemical remodeling events physically manifest as a significant increase in local matrix stiffness. In advanced atherosclerotic or fibrotic lesions, local tissue stiffness can increase to dozens or even over 50 kPa (43). This highly stiff, solid microenvironment provides key mechanical support for the long-term residency and pathological activation of SPP1^+^ macrophages, especially at the edge of the necrotic core with microcalcification.

In traditional immunology, vascular stiffening and disturbed flow are usually viewed as end products of the inflammatory response. However, with the development of mechanoimmunology, this one-way causal understanding is changing. Existing evidence shows that changes in the physical microenvironment also act as initiators driving disease progression (46). In a study using bovine aortic endothelial cells cultured on hydrogels, it was found that endothelial cells grown on more compliant matrices displayed increased elongation and tighter endothelial cell junctions, decreased activation, but enhanced nitric oxide production (47, 48). Conversely, these findings suggest that the progressive stiffening of the vascular wall intrinsically triggers endothelial dysfunction, thereby actively initiating the inflammatory cascade.

**Figure 2:**
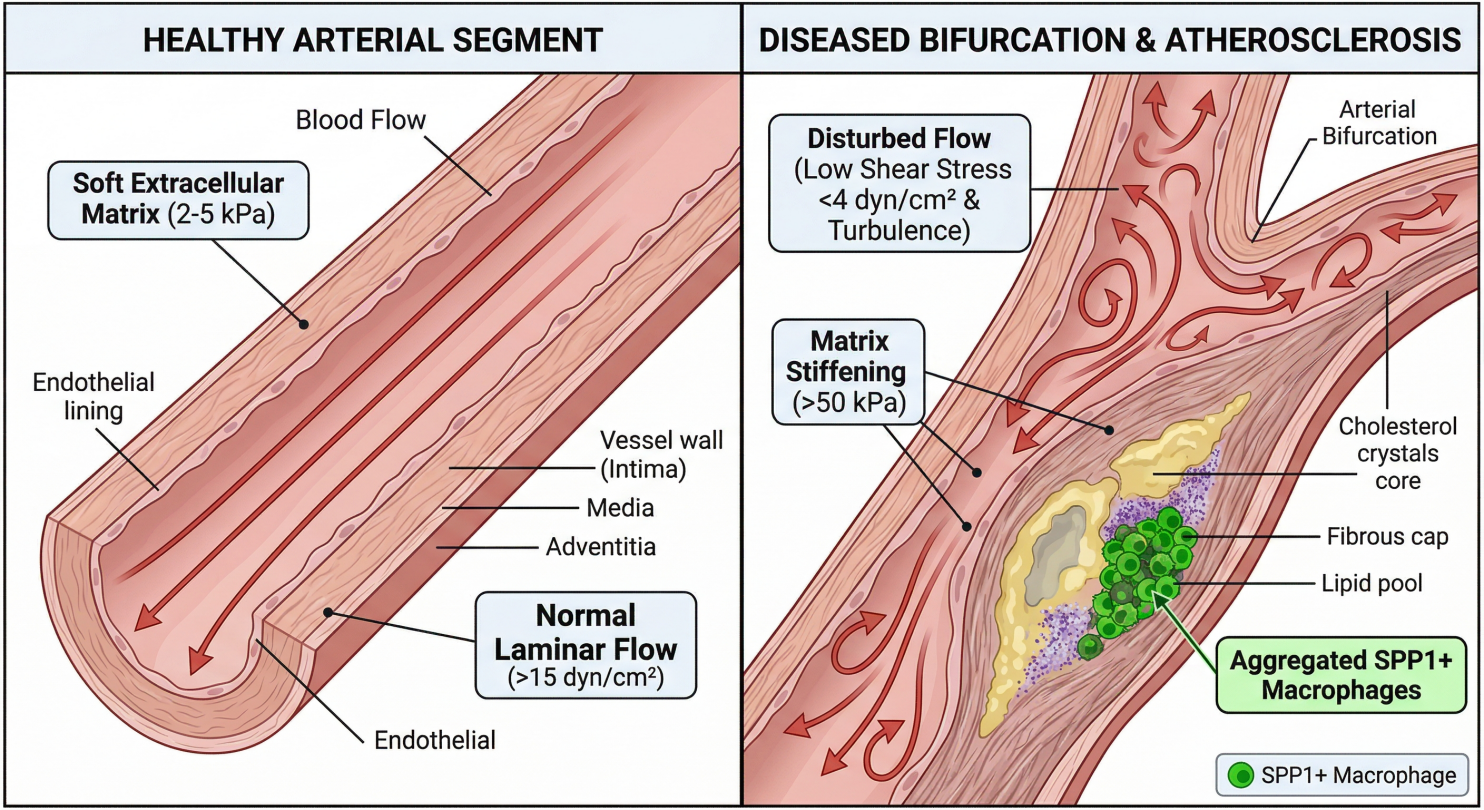
Spatial characteristics of the vascular microenvironment. (Left) Normal laminar flow and soft matrix in a healthy artery. (Right) Disturbed flow and matrix stiffening at the arterial bifurcation synergistically promote plaque formation and SPP1^+^ macrophages accumulation around the lipid core. (Created with BioGDP.com).

As spatiotemporally intersecting pathological mechanical cues, disturbed flow and abnormally increased matrix stiffness can directly mediate intracellular signaling in macrophages (30, 49). The superimposition of these two mechanical stimuli in specific anatomical areas, such as the subintima at arterial bifurcations, may provide a mechanistic explanation for the strict spatial limitations of SPP1^+^ macrophages. In this localized lesion microenvironment, physical stress is proposed to transcend its role as a mere structural scaffold, functioning instead as a key nonbiochemical signal source (22). This mechanical interplay is hypothesized to promote the adaptive transcriptional reprogramming observed in these pathogenic macrophages. (**Figure 2**).

### 2.2 Ttransmembrane integrins and cytoskeleton remodeling

Macrophages sense localized matrix stiffening, driven by collagen cross-linking and pathological remodeling, primarily through specific surface integrin heterodimers (50–52). Within this complex microenvironment, distinct integrin subtypes dictate the specificity of cell-extracellular matrix interactions. During the early stages of pathogenesis, the macrophage-enriched integrin α_M_β_2_(Mac-1/CD11b) mediates initial mechanical probing, while α_5_β_1_ preferentially binds to the fibronectin-rich provisional matrix (53, 54). As the disease progresses, the highly expressed α_v_β_3_ integrin assumes a dominant role by specifically recognizing and binding to the RGD motifs present in the SPP1 protein and other calcified matrix components (55, 56). In the soft matrix of physiological states, these integrin-ligand interactions are characteristically transient and exhibit low affinity. However, upon matrix stiffening, the amplified external mechanical resistance drives the allosteric activation and subsequent clustering of these integrins (52). This high-affinity state orchestrates the recruitment of various adaptor proteins, including focal adhesion kinase (FAK), paxillin, and Src family kinases, to the intracellular domain (57, 58). To effectively couple this physical anchoring at the membrane surface to the intracellular actomyosin cytoskeleton, structural linker proteins such as talin and vinculin serve as indispensable bridges (22, 59, 60). They physically connect the cytoplasmic tails of the integrins directly to F-actin filaments, thereby propagating external mechanical resistance deeply into the cellular interior. This physical transduction triggers actin polymerization and non-muscle myosin II-dependent contraction. The resulting confrontation between internal and external forces not only alters cellular morphology but also generates robust intracellular tension, establishing the fundamental physical prerequisite for subsequent mechanosensitive transcriptional regulation.

### 2.3 Mechanosensitive ion channels and primary cilia

Unlike integrins that primarily probe solid matrix mechanics, mechanosensitive ion channels like PIEZO1 mediate macrophage responses to dynamic physical stimuli such as flow shear stress and matrix stretching (61, 62). In the disturbed flow microenvironment at arterial bifurcations, abnormal shear stress increases local membrane tension in macrophages. This physical deformation triggers conformational changes and opening of the PIEZO1 channel, mediating a massive influx of calcium ions into the cell (63, 64). This transient calcium signal rapidly activates downstream effector molecules, notably calmodulin-dependent protein kinase II (CaMKII) and calcineurin (65–67). Subsequently, calcineurin induces the nuclear translocation of the nuclear factor of activated T cells (NFAT). In parallel, this calcium-driven cascade strongly intersects with the RhoA/ROCK pathway to augment intracellular cytoskeletal tension (68, 69). Ultimately, it is highly plausible that the synergistic integration of the calcineurin/NFAT signaling axis and RhoA/ROCK-mediated cytoskeletal contractility promotes YAP/TAZ nuclear activation. This coordinated cascade is hypothesized to provide a crucial mechanotransduction bridge linking PIEZO1 activation to the transcriptional upregulation of SPP1 and associated profibrotic genes, although the definitive direct causality in macrophages warrants further experimental validation.

In addition to transmembrane receptors, the sensing role of primary cilia in vascular mechanobiology is gradually gaining attention. While their mechanosensory functions are well characterized in endothelial cells, primary cilia represent an emerging and less-established apparatus in macrophages (70–72). When macrophages are exposed to disturbed flow in atherosclerosis-prone areas, abnormal shear stress can cause physical bending of the primary cilia. To understand the potential downstream cascades triggered by this deformation, researchers often draw parallels from well-established models. For instance, in the primary cilium of the renal collecting duct epithelium, physical deformation triggers the activation of the polycystin complex, particularly the polycystin-2 ion channel subunit (73). It is hypothesized that similar mechanisms, relying on microtubule network remodeling to transmit physical signals to the perinuclear region, might theoretically operate in vascular macrophages (74). Furthermore, substantial crosstalk likely exists between these hypothetical cilia-mediated signaling pathways and established integrin receptor networks (75). However, because downstream ciliary signaling varies profoundly among different cell types, these classical mechanisms derived from renal tubular epithelial cells cannot be directly extrapolated to immune cells without appropriate evidence. Currently, the precise molecular cascades linking primary cilia bending to macrophage transcriptional reprogramming are largely undefined. Therefore, the direct contribution of primary cilia to SPP1^+^ macrophage activation remains highly speculative and awaits rigorous experimental determination.

Crucially, these distinct mechanosensors do not operate in isolation but engage in extensive intracellular crosstalk to amplify physical signals. For example, integrin-dependent adhesion firmly anchors the cell to the extracellular matrix, which directly modulates local plasma membrane tension and thereby lowers the activation threshold for PIEZO1 channels. Conversely, the intracellular calcium influx driven by PIEZO1 profoundly enhances actomyosin contractility, which in turn reinforces integrin clustering and focal adhesion maturation (23, 76). This dynamic bidirectional interplay ensures that multiple mechanical cues converge synergistically to drive the pathogenic transcriptional reprogramming of macrophages.

The synergy of multiple mechanisms amplifies macrophage pathology in the complex local mechanical microenvironment (**Table 1**).

**Table 1:** Key macrophage mechanosensors and transduction pathways in the vascular microenvironment.

Mechanosensor	Primary physical stimuli	Physical/conformational response	Key downstream effectors	Strength of evidence
Integrins	Extracellular matrix stiffening (>50 kPa) and collagen cross-linking.	Allosteric activation in a stiffened matrix, leading to a high-affinity state, receptor clustering, and enhanced physical coupling with the actomyosin network.	Focal adhesion kinase (FAK), paxillin, and Src family kinases trigger the RhoA/ROCK pathway and YAP/TAZ activation.	Strongly Supported
PIEZO1 Channel	Disturbed flow shear stress and local membrane tension alterations.	Physical deformation triggers conformational changes and channel opening, mediating a massive influx of calcium ions (Ca2+).	Calmodulin-dependent kinase (CaMKII) and calcineurin/NFAT, intersecting with RhoA/ROCK contractility (hypothesized to link to SPP1 upregulation).	Moderately Supported (Direct causality to SPP1 awaits further experimental validation)
Primary Cilia	Disturbed flow (d-flow) and abnormal shear stress at arterial bifurcations.	Physical bending of the cilia and microtubule network remodeling to transmit physical signals to the perinuclear region.	Specific downstream targets and precise molecular cascades in macrophages remain largely undefined.	Emerging/Speculative

## 3 Intracellular mechanical signal transduction and transcriptional reprogramming of the SPP1^+^ macrophages

### 3.1 Nuclear translocation of YAP/TAZ and activation of target genes

Upon perceiving external physical cues through the aforementioned surface receptors, macrophages must propagate these mechanical signals inwards to the nucleus. The Hippo pathway effectors, YAP and TAZ, act as the ultimate molecular switches in this process. Under physiological conditions, such as a soft matrix or normal laminar flow, intracellular YAP and TAZ are primarily phosphorylated by upstream kinases, including large tumor suppressor 1 (LATS1) and large tumor suppressor 2 (LATS2). These phosphorylated proteins are subsequently sequestered in the cytoplasm for ubiquitin-mediated degradation, thereby preventing their nuclear translocation (77, 78). When cells encounter a pathological mechanical microenvironment, physical tension disrupts these canonical biochemical inhibition pathways (79).

For instance, a local matrix stiffness exceeding 50 kPa or the presence of abnormally disturbed flow effectively activates the Ras homolog family member A (RhoA) and Rho-associated coiled-coil containing protein kinase (ROCK) signaling pathways via integrins and PIEZO1 channels (80). RhoA activation promotes the polymerization of fibrillar actin (F-actin) and the contraction of nonmuscle myosin II (NMII). This assembles a contractile actomyosin network inside the cell. The resulting cytoskeletal tension mediates the flattening or stretching of the nucleus. It also alters the conformation of nuclear pore complexes (NPCs) through physical traction, increasing nuclear pore permeability. Changes in cytoskeletal tension cause the dephosphorylation of YAP and TAZ in the cytoplasm, followed by significant nuclear translocation (81–83). This provides the spatial basis for these two transcriptional coactivators to participate in genomic regulation.

**Figure 3:**
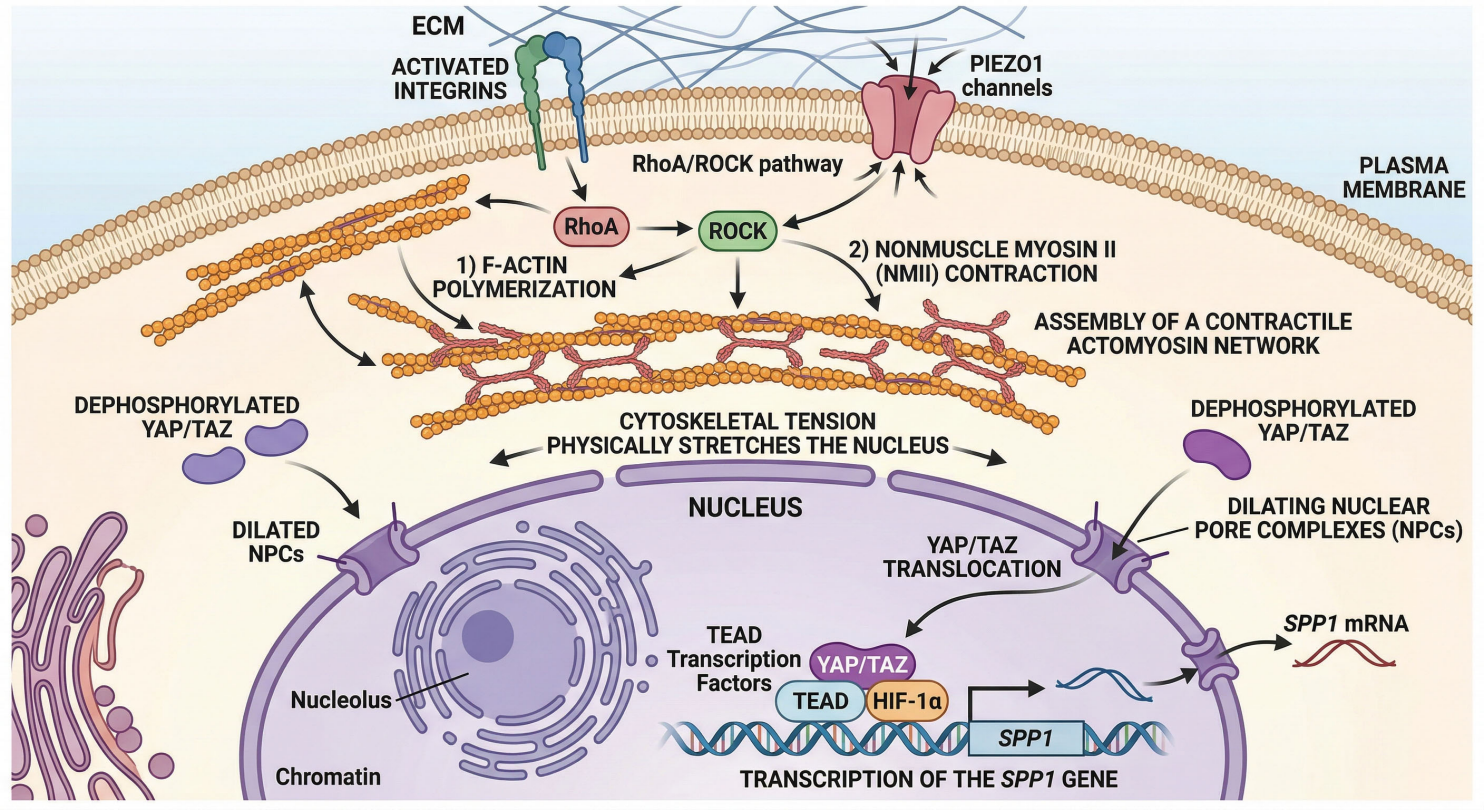
Mechanism of mechanotransduction-induced *SPP1* expression. ECM stiffness activates integrins and PIEZO1, triggering the RhoA/ROCK pathway. This results in actomyosin contraction that physically stretches the nucleus, allowing YAP/TAZ to enter through dilated nuclear pores. Inside, YAP/TAZ works with TEAD and HIF-1α to upregulate *SPP1* transcription, promoting macrophage accumulation in plaques. (Created with BioGDP.com).

The YAP and TAZ proteins lack a direct DNA-binding domain. The transcriptional activation of these genes highly depends on the synergistic recruitment of specific nuclear transcription factors (84). After nuclear translocation, YAP/TAZ mainly bind with TEAD (TEA domain) family transcription factors through their core binding motifs to form a heterodimeric complex (the YAP/TAZ-TEAD complex) (85–87). Recent chromatin immunoprecipitation sequencing (ChIP-seq) data have shown that in macrophages exposed to pathological matrix stiffness or abnormal shear stress, this complex binds to promoter regions of classic pro-proliferative and antiapoptotic genes (88). More importantly, it is significantly enriched in the regulatory sequences of a series of profibrotic and metabolic remodeling-related pathogenic genes. The assembly of this transcription factor complex constitutes a key regulatory node for the response of the macrophage genome to external mechanical stimuli. It achieves precise conversion from macroscopic physical forces to microscopic gene transcription instructions (89) (**Figure 3**).

### 3.2 Crosstalk between mechanical tension and hypoxic metabolic pathways

Crucially, in the complex microenvironment of advanced arterial lesions, mechanical stress does not operate in isolation but exhibits profound crosstalk with severe local hypoxia. Elevated matrix stiffness and heightened cytoskeletal tension can directly stimulate the phosphoinositide 3-kinase (PI3K)/protein kinase B (AKT) signaling cascade, which subsequently stabilizes hypoxia-inducible factor 1-alpha (HIF-1α) even under normoxic or mildly hypoxic conditions (90, 91). Conversely, accumulated HIF-1α physically interacts with YAP/TAZ, impeding their degradation and reinforcing their nuclear retention. This intricate mechano-metabolic coupling synergistically amplifies the transcription of *SPP1*. Furthermore, evidence derived predominantly from tumor-associated macrophage and fibrotic tissue models indicates that extracellular SPP1 can engage its surface receptor CD44 to trigger a secondary, sustained wave of PI3K/AKT activation (92, 93). While direct *in vivo* validation within atherosclerotic plaques is still emerging, this extrapolated signaling axis theoretically entrenches the survival and pathogenic reprogramming of SPP1^+^ macrophages.

To orchestrate this complex mechano-metabolic coupling, the Hippo pathway effector YAP/TAZ operates as a highly specific mechanosensitive hub within vascular lesions. In response to altered matrix stiffness, the nuclear retention of YAP/TAZ directly dictates the fibrotic reprogramming required for arterial remodeling. By binding to TEAD and cooperating intimately with stabilized HIF-1α, YAP/TAZ explicitly drives the robust transcription of *SPP1 (*94), thereby seamlessly connecting extracellular physical forces to localized immune-mediated vascular deterioration (95–97).

### 3.3 Transcriptional characteristics and spatial colonization of SPP1^+^ macrophages

The macroscopic mechano-metabolic coupling orchestrated by YAP/TAZ ultimately manifests as profound micro-level transcriptional reprogramming. With the advent of high-resolution single-cell transcriptomics, the heterogeneity of intravascular macrophages has been reconstructed, revealing the precise transcriptomic signature of this mechanosensitive adaptation (98). Cross-species evidence has shown that SPP1^+^ macrophages play a key role in disease progression, as shown in both mouse models and clinical human carotid plaques (99, 100). Exhibiting a unique transcriptional profile, this highly conserved SPP1^+^ macrophage subpopulation is specifically enriched in triggering receptor expressed on myeloid cells 2 (TREM2), cluster of differentiation 9 (CD9), and various cathepsins, including cathepsin D (CTSD), across different species (98, 99, 101).

Distinct from the transient proinflammatory monocyte-derived macrophages observed in early atherogenesis, SPP1^+^ macrophages significantly downregulate typical antigen-presenting genes, such as the major histocompatibility complex class II (MHC-II). Instead of executing classical immune defense functions, this specific transcriptional reprogramming redirects their cellular machinery toward extensive lipid handling, heightened lysosomal activity, and profound interactions with the extracellular matrix (101). Consequently, they transition into core effector cells that orchestrate deep structural remodeling of the local tissue (102).

Identifying the spatial localization of cells within lesions is essential for revealing their pathological functions. Spatial transcriptomics and multiplex immunofluorescence imaging revealed that SPP1^+^ macrophages are not randomly scattered throughout the vascular wall. They show high spatial heterogeneity and regional clustering (103, 104). As summarized in **Table 2**, this highly specialized pathological niche is characterized by severe hypoxia, massive lipid overload, necrotic core-associated stress, progressive local matrix stiffening, microcalcification, disturbed flow-induced endothelial activation, and steep inflammatory chemokine gradients (105, 106). Driven by this extreme microenvironment, SPP1^+^ macrophages function not merely as passive markers of disease progression, but rather as active determinants that dictate plaque vulnerability (107).

**Table 2:** Key microenvironmental factors driving SPP1^+^ macrophage enrichment and persistence.

Category	Microenvironmental factor	Pathological contribution to the SPP1^+^ niche
Hemodynamic & Mechanical Cues	Disturbed flow-induced endothelial activation	Initiates early subintimal leakage, promotes leukocyte recruitment, and triggers early mechanosensitive signaling cascades.
Hemodynamic & Mechanical Cues	Local matrix stiffening & microcalcification	Generates robust extracellular resistance, triggering integrin clustering and YAP/TAZ-dependent SPP1 transcription.
Metabolic & Chemical Stress	Severe localized hypoxia	Stabilizes HIF-1α, which intimately synergizes with mechanotransduction to reinforce pathogenic survival and persistence.
Metabolic & Chemical Stress	Massive lipid overload	Drives continuous phagocytosis, ultimately overwhelming lysosomal capacity and promoting foam cell formation.
Tissue Damage Signals	Necrotic core-associated stress	Provides abundant apoptotic debris, overloads efferocytosis, and sustains a microenvironment of chronic localized injury.
Immunological Drivers	Inflammatory chemokine gradients	Directs the precise spatial migration and targeted regional clustering of macrophages deep within the advancing lesion.

Furthermore, they often colonize specific hemodynamically susceptible sites, such as arterial bifurcations (107). This strict spatial limitation strongly suggests that local specific microenvironmental cues, such as local mechanical stress and hypoxia, play an irreplaceable decisive role. This action acts alongside classical soluble chemokines to induce the targeted recruitment and long-term residency of SPP1^+^ macrophages.

## 4 Pathogenic mechanical positive feedback loop and translational medicine intervention

### 4.1 Lipid metabolism collapse and local matrix remodeling

SPP1^+^ macrophages residing in the core plaque area exhibit a highly context-dependent role during the pathological evolution of the arterial wall (108). At the level of lipid metabolism, the high expression of various lipid scavenger receptors initially reflects an adaptive, foam-cell-like protective state. This allows them to efficiently phagocytose oxidized low-density lipoprotein (oxLDL) and clear apoptotic cell debris via efferocytosis (109). However, as the disease progresses to advanced stages, extreme microenvironmental stress and continuous lipid influx severely overload the lysosomal degradation system of SPP1^+^ macrophages (16, 110). Only at this late stage does this adaptive state transition into a highly pathogenic phenotype, causing significant impairment of efferocytosis. This ultimate collapse of local lipid metabolism triggers secondary necrosis (111). With the release of massive amounts of intracellular proinflammatory substances, the necrotic core of the lesion constantly expands. This causes irreversible tissue deterioration (112, 113).

In addition to disrupting lipid homeostasis, SPP1^+^ macrophages strongly influence the pathological remodeling of the ECM. Functionally, the SPP1 protein acts as a pivotal matricellular mediator, driving pro-fibrotic cascades and accelerating vascular calcification within the vessel wall (114). Moreover, this macrophage subpopulation secretes targeted matrix metalloproteinase family members, particularly MMP-9 and MMP-12 (115). Importantly, matrix degradation is a highly collaborative process; rather than acting alone, SPP1^+^ macrophages work in concert with MMPs derived from surrounding vascular smooth muscle cells (VSMCs), endothelial cells, and neutrophils to degrade the normal elastic fiber network that maintains vascular tone (2). Furthermore, they widely activate VSMCs and fibroblasts through paracrine pathways. This induces massive synthesis and cross-linking of prestiffening type I collagen. This synergistic interplay between the collapse of lipid homeostasis and the imbalance in ECM synthesis and degradation significantly compromises vascular wall compliance (17, 116). Consequently, this profoundly elevates the clinical risk of atherosclerotic plaque rupture. (**Table 3**).

**Table 3:** Pathological phenotypes of SPP1^+^ macrophages.

Phenotypic dimension	Specific characteristics	Pathological consequences
Transcriptomic Profile	Significantly enriched in TREM2, CD9, CTSD (Cathepsin D), and SPP1, alongside high levels of lipid scavenger receptors and matrix metalloproteinases (MMPs).	Overcomes standard immune defense functions, transforming into core effector cells that drive deep structural remodeling of local tissues.
Immunity & Antigen Presentation	Significant downregulation of typical antigen-presenting genes, such as MHC-II.	Loses the classical pro-inflammatory and antigen-presenting functions characteristic of early monocyte-derived macrophages.
Lipid Metabolism	Continuous phagocytosis of oxidized low-density lipoprotein (oxLDL) and apoptotic debris; severe overload of the lysosomal degradation system leads to impaired efferocytosis.	Triggers the collapse of local lipid metabolism, resulting in foam cell formation, secondary necrosis, and the continuous expansion of the necrotic core.
ECM Remodeling	Secretion of MMPs degrades the elastic fiber network; paracrine signaling activates VSMCs/fibroblasts to synthesize type I collagen. Secreted SPP1 actively mediates collagen cross-linking and microcalcification.	Severely compromises vascular compliance and significantly increases local matrix stiffness (>50 kPa), profoundly elevating the risk of plaque rupture.

### 4.2 Pathomechanical closed-loop between the microenvironment and cells

While we propose an integrated pathomechanical closed-loop, distinguishing between firmly established regulatory steps and extrapolated concepts is essential for scientific accuracy. Direct experimental evidence strongly supports several distinct elements of this cascade. Specifically, the activation of YAP/TAZ in response to matrix stiffness, the resulting transcriptional upregulation of *SPP1*, and the subsequent role of SPP1 in driving ECM microcalcification are well documented (117, 118). In contrast, an uninterrupted *in vivo* mechanotransduction axis that directly links initial PIEZO1 or ciliary activation to the full SPP1^+^ pathogenic phenotype in atherosclerosis lacks definitive *in vivo* proof. Much of this continuous sequence is currently inferred from *in vitro* macrophage cultures and parallel signaling pathways identified in fibroblasts. Therefore, the self-amplifying positive feedback loop described in the following sections should be viewed as a synthetic hypothesis. We have constructed this conceptual model by bridging multidisciplinary findings, aiming to provide a theoretical foundation that awaits rigorous validation through future *in vivo* lineage-tracing and targeted mechanosensor-knockout studies.

In traditional pathology, the effects of hemodynamics (fluid shear stress) and matrix stiffness (solid mechanics) on cells are often discussed in isolation. However, the physical microenvironment of the vascular system is highly integrated and dynamically interactive. Emerging mechanobiological evidence shows that during the progression of atherosclerosis and tissue fibrosis, disturbed flow and matrix stiffening are not independent pathological events. Instead, through macrophages as key nodes, they form a mutually promoting and self-amplifying positive feedback loop (119). This closed-loop mechanism accelerates the pathological process of local vascular injury, pushing the lesion toward continuous deterioration.

The starting point of this pathological cycle usually originates from hemodynamic disturbances at arterial bifurcations or curves. Long-term exposure to a low-level, oscillatory shear stress (d-flow) environment induces increased endothelial permeability and the expression of proinflammatory adhesion molecules (120, 121). It further initiates early mechanical remodeling of the subintimal ECM. At this stage, early macrophages recruited by disturbed flow and residing locally sense blood flow signals through primary cilia and PIEZO1 channels. This process leads to initial cytoskeletal reorganization. It also prompts macrophages to secrete MMP and transforming growth factor-beta (TGF-β) (122, 123). These factors act synergistically to degrade the originally regular elastin network under the intima. They stimulate adjacent smooth muscle cells and fibroblasts to synthesize prestiffening collagen, inducing an initial increase in local matrix stiffness.

With continuous collagen deposition and cross-linking, local matrix stiffness gradually deviates from the physiological range. At this stage, changes in the mechanical properties of solid tissue play an important role in driving pathological progression. In a highly stiff microenvironment, surface integrins on macrophages undergo high-affinity clustering. This induces a significant increase in cytoskeletal tension. This physical stress promotes the migration and accumulation of YAP/TAZ in the nucleus by altering the physical conformation of nuclear pore complexes. Sustained nuclear localization of YAP/TAZ mediates the transcriptional reprogramming of macrophages toward the SPP1^+^ phenotype (60). It also enhances the antiapoptotic ability of this subpopulation. These metabolic and transcriptional changes enable SPP1^+^ macrophages to adapt to the extreme microenvironment at the edge of the necrotic core and maintain pathological activation.

SPP1 plays a core role in maintaining vascular pathological mechanical homeostasis. After release into the ECM, SPP1 uses its RGD (Arg-Gly-Asp) motif sequence to mediate pathological cross-linking of collagen fibers, enhancing the solid mechanical strength of the tissue (124, 125). Furthermore, SPP1-mediated microcalcification significantly increases matrix stiffness, destroying the physiological compliance of the vascular wall. This mechanism embodies a clear mechanical interactive logic: initial matrix remodeling triggered by abnormal blood flow locks in the pathogenic macrophage phenotype via the integrin and YAP/TAZ pathways.

**Figure 4:**
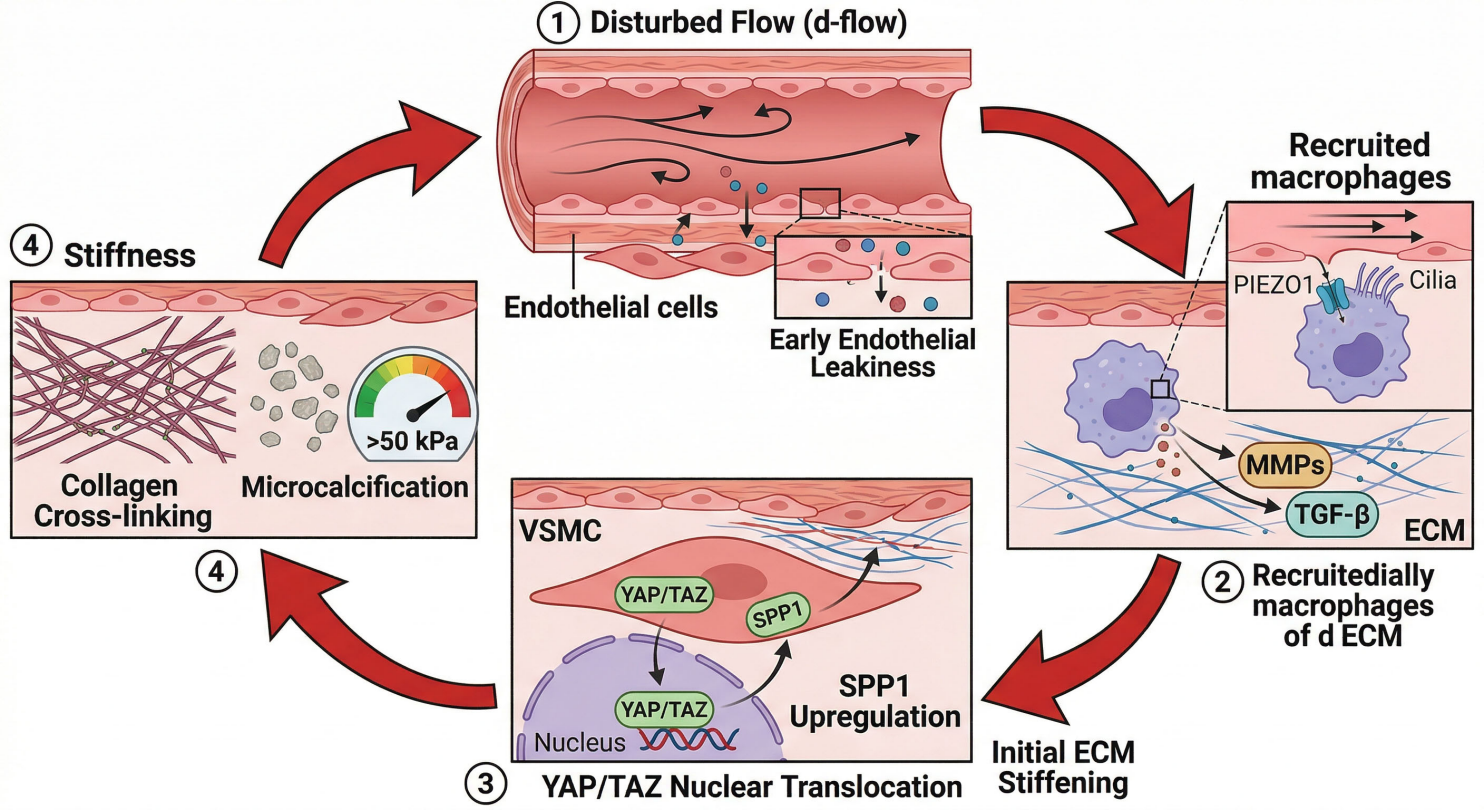
Pathological cycle of disturbed flow-driven vascular stiffening. D-flow triggers endothelial leakiness and macrophage recruitment, initiating early ECM stiffening via MMPs and TGF-β secretion, which further activates YAP/TAZ nuclear translocation and SPP1 upregulation in VSMCs, ultimately exacerbating vascular stiffness through collagen cross-linking and microcalcification. (Created with BioGDP.com).

The subsequently produced SPP1 effector molecules exacerbate matrix stiffening, providing continuous power for this positive feedback loop (8). This cycle, which is initiated by physical stress and amplified by biochemical products, causes the local lesion to break free from physiological regulation. It becomes the core physical–biological mechanism driving the transformation of atherosclerosis into complex plaques or severe fibrosis. (**Figure 4**).

### 4.3 Experimental verification of the biological bionic platform and the genetic engineering model

Elucidating these theoretical mechanisms requires systematic validation through sophisticated biomimetic models. Rigorous verification is crucial for establishing the actual pathological relevance of this mechanosensitive feedback loop.

*In vitro*, utilizing standardized monocyte cell lines, such as Tohoku Hospital Pediatrics-1 (THP-1) cells, in conjunction with tunable physical microenvironment platforms, is a cornerstone strategy for dissecting macrophage mechanosensing mechanisms (126, 127). By seeding differentiated THP-1-derived macrophages onto polyacrylamide (PAA) or specialized hydrogel substrates with a gradient Young’s modulus, researchers can precisely recapitulate the mechanical properties of the matrix in both physiological (2–5 kPa) and pathological stiffening (>50 kPa) states. Furthermore, integrating parallel-plate flow chamber systems enables the application of defined patterns of fluid shear stress (128). The synergy of immunofluorescence and live-cell imaging facilitates the real-time visualization of dynamic mechanobiological events (129), including PIEZO1-mediated calcium influx, cytoskeletal structural rearrangement, and YAP/TAZ nuclear translocation. However, it should be noted that THP-1 cells are an immortalized monocytic leukemia-derived cell line and may not fully recapitulate the behavior of primary or plaque-resident macrophages. Therefore, we recommend that findings obtained from such cell lines be further validated in primary macrophage systems such as mouse bone marrow-derived macrophages or human monocyte-derived macrophages.

**Figure 5:**
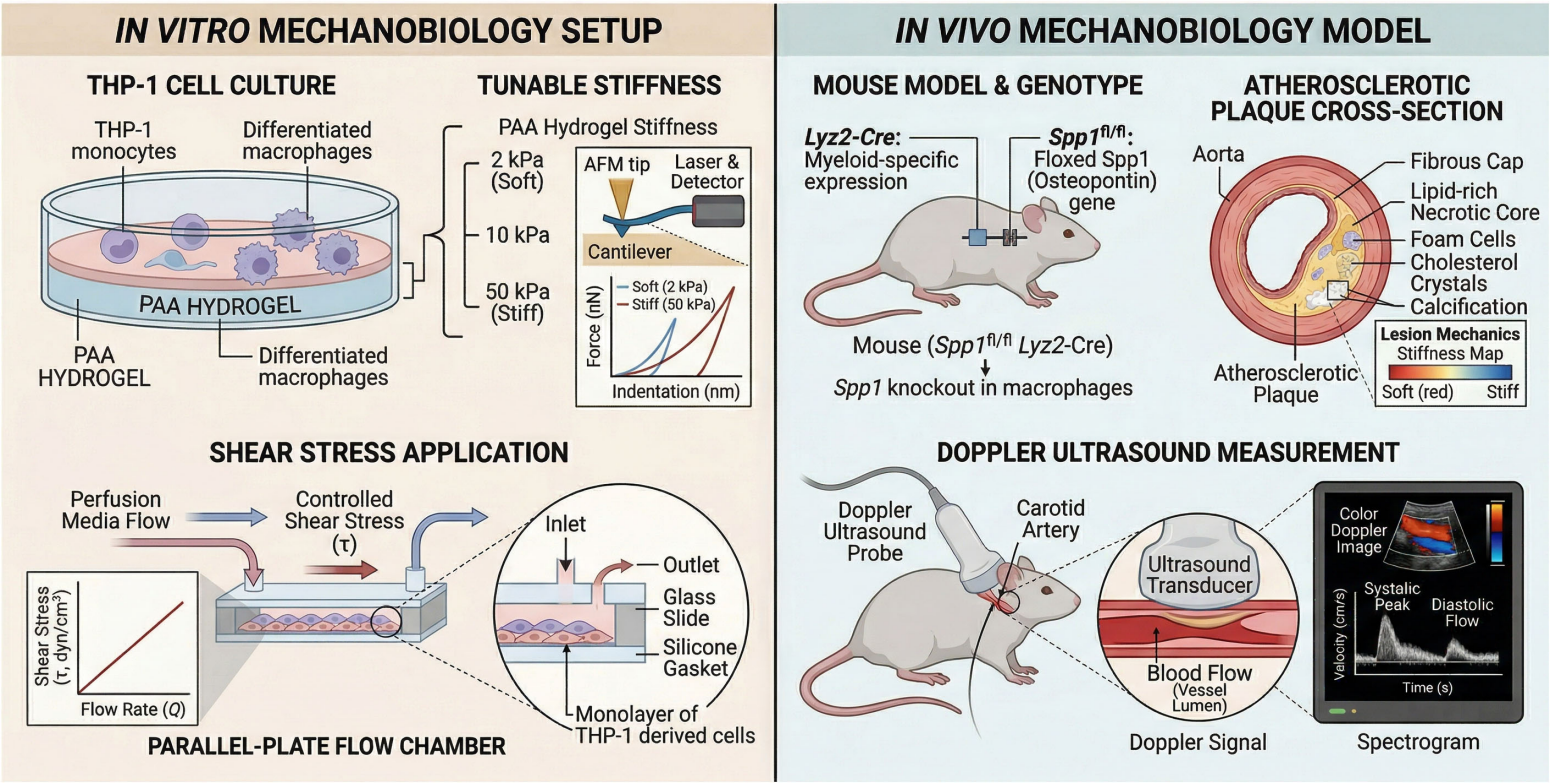
Schematic of the *in vitro* and *in vivo* mechanobiology experimental setup. (Left)THP-1 cells are cultured on tunable PAA hydrogels (2–50 kPa) to simulate substrate stiffness, with a parallel-plate flow chamber used to apply fluid shear stress. Stiffness is validated via AFM. (Right)A myeloid-specific knockout mouse model (*Spp1*^flox/flox^ *Lyz2*-Cre) is used to study atherosclerotic plaques. Doppler ultrasound is employed to measure hemodynamic flow within the vessel. (Created with BioGDP.com).

While *in vitro* systems provide high-resolution molecular perspectives, verifying the mechanical synergistic positive feedback loop still requires a complex *in vivo* physiological environment. Wild-type C57BL/6J mice, after undergoing disturbed flow surgery or high-fat induction, can simulate vascular mechanical remodeling features in lesions such as atherosclerosis and abdominal aortic aneurysms (AAAs) well (130, 131). To further verify the role of *SPP1* in this mechanical regulatory pathway, global knockout (*Spp1*^-/-^) models are widely used to assess the mitigation of vascular microcalcification and fibrosis (17, 132). Considering that cells such as smooth muscle cells in the microenvironment can also secrete osteopontin, constructing macrophage-specific conditional knockout models is important to clarify cell-specific functions. By crossing *Spp1*^flox/flox^ mice with *Lyz2*-Cre mice expressing specifically the myeloid lineage, researchers can establish an *Spp1*^flox/flox^ *Lyz2*-Cre conditional knockout model. Nevertheless, the Lyz2-Cre driver targets broad myeloid populations, including neutrophils and monocytes, rather than being strictly macrophage-specific (133). In future studies, employing more selective Cre drivers such as Cx3cr1-CreER or Ms4a3-Cre would provide greater cell-type specificity for clarifying macrophage-specific functions (134, 135). Using such genetic tools, combined with ultrasound Doppler hemodynamic analysis and atomic force microscopy (AFM) to determine matrix stiffness, researchers can effectively analyze the contribution of macrophage-derived SPP1 to the response to changes in shear stress and matrix stiffening (136). These findings further clarify its regulatory effect on AAA deterioration and increased plaque vulnerability. (**Figure 5**).

### 4.4 Local targeted therapy for the mechanotransduction pathway

The interplay between the local physical microenvironment and macrophage mechanotransduction represents a key driver of vascular pathology. Shifting therapeutic strategies from traditional systemic modulation, such as lipid-lowering or anti-inflammatory therapies, toward localized mechanoimmunomodulation offers a promising approach to treating arterial diseases. By intercepting the pathways through which macrophages sense and respond to aberrant mechanical stimuli, it is possible to attenuate the pathological induction of SPP1 and the subsequent maladaptive matrix remodeling. This approach targets the underlying biomechanical triggers of disease, potentially mitigating the progression of lesions that are refractory to conventional treatments.

A direct strategy to intervene in mechanotransduction is to inhibit the ability of macrophages to sense abnormal stiffness and blood flow. PIEZO1, a mechanosensitive ion channel, is becoming an important pharmacological target. Preclinical studies have shown that the use of specific peptide inhibitors such as GsMTx4 to block PIEZO1 can significantly reduce calcium signals in macrophages, thereby inhibiting their transition to a profibrotic phenotype (137, 138). Additionally, intervening in physical adhesion between cells and stiffened matrices holds therapeutic potential (139). Integrin blockers (such as αvβ3 or α5β1 inhibitors) can intervene in this process. Using small-molecule drugs to inhibit FAK, a key downstream kinase of integrins, can drastically reduce cytoskeletal contractile tension (140). This disrupts mechanical transmission between macrophages and the ECM, thereby delaying microcalcification in the lesion area.

Directly inhibiting YAP/TAZ can block the core link of mechanical force transmission to the genome. Since various upstream physical stimuli eventually converge on this nuclear target, inhibiting YAP/TAZ has a broad-spectrum anti-pathological remodeling effect. Verteporfin, a small-molecule drug that acts as a YAP-TEAD interaction disruptor, has significant intervention effects *in vitro (*141). In macrophages exposed to high matrix stiffness and treated with verteporfin, *SPP1* expression and the matrix cross-linking capacity are significantly inhibited. However, YAP/TAZ also plays physiological roles in maintaining endothelial barrier function and tissue homeostasis. Therefore, preserving its physiological repair function while inhibiting pathogenic transcription is the main challenge for this strategy.

Despite promising preclinical observations, a critical assessment of translational feasibility reveals significant clinical limitations. The realistic clinical potential of systemic pharmacological agents such as GsMTx4 and Verteporfin is heavily restricted by poor pharmacokinetics, low delivery efficiency, and severe dose-limiting toxicity. Advancing the clinical translation of mechanomedicine requires overcoming potential off-target effects. Because physical tension widely exists across organ systems, systemic administration of PIEZO1 or YAP inhibitors may cause adverse reactions (142). Specifically, physiological mechanotransduction is fundamentally required for the homeostasis of healthy endothelial cells, vascular smooth muscle cells, and various other tissues. Indiscriminate systemic inhibition of these core mechanosensors could catastrophically interfere with normal vascular tone regulation. Beyond broad systemic toxicities, the blunt inhibition of universal mechanosensors poses a severe risk to local tissue homeostasis. Within healthy vascular segments, basal mechanotransduction is required for resident macrophages to perform routine efferocytosis, thereby efficiently clearing apoptotic cells and preventing secondary necrosis. Furthermore, during the critical stages of plaque stabilization, non-selective targeting of YAP/TAZ or PIEZO1 might inadvertently impair the physiological functions of beneficial macrophage subsets and smooth muscle cells that are indispensable for synthesizing the protective fibrous cap (143). Consequently, systemic mechano-inhibition could paradoxically increase plaque vulnerability by disrupting these delicate structural repair mechanisms.

To overcome these critical translational barriers and mitigate systemic toxicity, employing advanced lesion-targeted nanodelivery strategies is strictly required. Therefore, highly customized nanotargeted delivery systems are gradually being developed. Lipid nanoparticles (LNPs) modified with ligands for specific macrophage surface receptors, such as CD36 or the mannose receptor, can achieve precise drug enrichment in macrophages at local lesions. Moreover, smart nanocarriers responsive to specific disturbed shear stress or high-MMP environments combine mechanoresponsive materials with immunomechanical inhibitors. This dual-targeting strategy can increase the local drug concentration. This approach provides a viable engineering solution to reduce systemic toxicity and achieve precise vascular mechano-immunomodulation. By specifically accumulating in the pathological microenvironment, these targeted nanocarriers can maximize the local therapeutic efficacy while safely preserving systemic mechanobiological homeostasis.

## 5 Discussion

The spatial distribution of vascular lesions is intrinsically linked to specific local mechanical microenvironments. This review synthesizes current evidence to illustrate how physical cues, specifically disturbed hemodynamics and matrix stiffening, act as crucial stimuli in arterial remodeling. Rather than merely functioning as passive bystanders, macrophages sense these environmental changes, either indirectly through endothelial barrier dysfunction or directly via specific mechanosensors.

Current experimental evidence strongly supports that mechanosensing via integrins translates macroscopic physical forces into intracellular cytoskeletal tension, which dictates the transcriptional reprogramming of target genes via the YAP/TAZ signaling axis. This established pathway directly upregulates *SPP1*. Conversely, the direct contribution of emerging mechanosensors, such as PIEZO1 ion channels and primary cilia, to the full SPP1^+^ pathogenic phenotype *in vivo* remains largely speculative and requires further rigorous experimental validation.

Functionally, SPP1^+^ macrophages exhibit a context-dependent role. They initially display adaptive lipid-handling capabilities before extreme microenvironmental stress leads to lysosomal overload and secondary necrosis. Furthermore, through collaborative matrix degradation with other vascular cells and SPP1-mediated collagen crosslinking, this macrophage subset significantly increases local matrix stiffness. Based on these multidisciplinary syntheses, we propose a hypothetical pathomechanical positive feedback loop: initial mechanical stress drives the pathogenic transition of macrophages, which in turn secrete effectors that further exacerbate tissue rigidity.

While targeting these mechanotransduction nodes presents a theoretical framework for managing macrophage-mediated arterial diseases, systemic pharmacological inhibition faces severe translational limitations, including off-target effects and catastrophic interference with physiological mechanohomeostasis. Therefore, future clinical applications must rely on the development of highly specific, lesion-targeted nanodelivery systems to safely and effectively disrupt this pathological mechanical cycle.
